# Machine Learning for Quality Control in the Food Industry: A Review

**DOI:** 10.3390/foods14193424

**Published:** 2025-10-04

**Authors:** Konstantinos G. Liakos, Vassilis Athanasiadis, Eleni Bozinou, Stavros I. Lalas

**Affiliations:** 1 Department of Electrical and Computer Engineering, University of Thessaly, Sekeri Street, 38334 Volos, Greece; kliakos@uth.gr; 2 Department of Food Science and Nutrition, University of Thessaly, Terma N. Temponera Street, 43100 Karditsa, Greece; vaathanasiadis@uth.gr (V.A.); empozinou@uth.gr (E.B.)

**Keywords:** machine learning, food quality control, defect detection, smart packaging, explainable AI, ingredient optimization, predictive analytics, food traceability, Industry 4.0, sensor-based inspection

## Abstract

The increasing complexity of modern food production demands advanced solutions for quality control (QC), safety monitoring, and process optimization. This review systematically explores recent advancements in machine learning (ML) for QC across six domains: Food Quality Applications; Defect Detection and Visual Inspection Systems; Ingredient Optimization and Nutritional Assessment; Packaging—Sensors and Predictive QC; Supply Chain—Traceability and Transparency and Food Industry Efficiency; and Industry 4.0 Models. Following a PRISMA-based methodology, a structured search of the Scopus database using thematic Boolean keywords identified 124 peer-reviewed publications (2005–2025), from which 25 studies were selected based on predefined inclusion and exclusion criteria, methodological rigor, and innovation. Neural networks dominated the reviewed approaches, with ensemble learning as a secondary method, and supervised learning prevailing across tasks. Emerging trends include hyperspectral imaging, sensor fusion, explainable AI, and blockchain-enabled traceability. Limitations in current research include domain coverage biases, data scarcity, and underexplored unsupervised and hybrid methods. Real-world implementation challenges involve integration with legacy systems, regulatory compliance, scalability, and cost–benefit trade-offs. The novelty of this review lies in combining a transparent PRISMA approach, a six-domain thematic framework, and Industry 4.0/5.0 integration, providing cross-domain insights and a roadmap for robust, transparent, and adaptive QC systems in the food industry.

## 1. Introduction

QC is a cornerstone of the global food industry, ensuring safety, regulatory compliance, and consumer trust in increasingly complex and competitive markets. Effective QC systems mitigate risks associated with contamination, adulteration, fraud, and process variability—hazards that can cause widespread foodborne illness, economic loss, and reputational damage if left unchecked [1]. Traditional QC approaches, such as manual inspection, laboratory testing, and batch sampling, have been indispensable for decades but are often reactive, labor-intensive, and limited in scalability, responsiveness, and precision, particularly within the context of globalized supply chains and evolving consumer demands [1,2].

Internationally recognized standards—such as Hazard Analysis and Critical Control Points (HACCP), Good Manufacturing Practices (GMP), ISO 9001, and ISO 22000—form the backbone of regulatory QC frameworks administered by bodies including the U.S. Food and Drug Administration (FDA), European Food Safety Authority (EFSA), and the Codex Alimentarius Commission [2]. These frameworks emphasize preventive control, risk-based evaluation, and traceability, requiring systematic monitoring from raw materials through processing, packaging, and distribution. Nevertheless, challenges persist in harmonizing requirements across jurisdictions, combating increasingly sophisticated food fraud, and ensuring transparency throughout extended supply chains [3].

Recent advances in sensing technologies (e.g., hyperspectral imaging, biosensors, Internet-of-Things networks) and the proliferation of high-volume, high-velocity production and environmental data have created fertile ground for a paradigm shift in QC methodology. Within this context, ML—a branch of artificial intelligence (AI)—has emerged as a transformative enabler of real-time, predictive, and adaptive QC [4]. Unlike static, rule-based systems, ML algorithms can learn from historical and streaming data to identify complex, nonlinear patterns, optimize operational parameters, and forecast quality outcomes before defects occur [4,5].

Applications of ML in food QC span a wide spectrum: (1) real-time, non-destructive quality assessment to complement or replace destructive laboratory methods [5]; (2) high-throughput visual inspection using computer vision to detect surface defects and classify products by grade or specification [5]; (3) predictive and preventive modeling for shelf-life estimation, microbial growth prediction, and process deviation alerts [4]; (4) multi-modal data integration, combining imaging, spectral, chemical, and sensor data for comprehensive quality profiling [4].

Algorithmic approaches include traditional supervised classifiers (e.g., support vector machines (SVMs), decision trees, k-nearest neighbors (KNN), unsupervised and semi-supervised anomaly detectors, ensemble learning methods) and, increasingly, deep learning (DL) architectures such as convolutional neural networks (CNNs) and generative adversarial networks (GANs), which excel in high-dimensional image and sensor data fusion [4,5]. These technologies are increasingly being embedded within the broader framework of Industry 4.0, integrating ML with IoT, blockchain, and digital twin technologies to enable interconnected, self-optimizing, and transparent food production systems [6].

The present review systematically analyses the role of ML in enhancing QC processes across the food industry. Drawing upon recent literature, it categorizes applications by domain, synthesizes methodological trends, and evaluates opportunities and challenges in moving from laboratory prototypes to industrial implementation. In doing so, it aims to provide researchers, practitioners, and policymakers with a clear understanding of how ML can advance safe, sustainable, and future-ready food quality management.

This review differs from prior work by applying a PRISMA-based systematic selection process, introducing a six-domain thematic classification for ML applications in food QC, and emphasizing metric alignment for cross-study comparability. It also integrates emerging Industry 4.0/5.0 concepts into the QC framework, offering a broader and more interconnected perspective than existing reviews.

## 2. ML—Methods in the Service of Food QC

### 2.1. Scope and Relevance

In food QC, the central question is not what ML is, but which ML approach most effectively addresses a QC problem under industrial constraints such as speed, non-destructive testing, traceability, and regulatory compliance [1]. Accordingly, this section introduces only those methods that were identified in our review and applies them directly to QC tasks and data modalities, including imaging, spectroscopy, inline sensing, environmental monitoring, and supply chain signals. The description is therefore framed within the six-domain taxonomy established in Section 3 and aligned with the PRISMA selection process [2].

### 2.2. Vision-Based Methods for Defect Detection and Grading

CNNs are the prevailing models for visual QC because they automatically extract relevant features from RGB or hyperspectral images [7,8]. They are particularly effective in the (i) detection of defects such as cracks, bruises, or contamination in eggs, gummies, and printed confectionery [5]; (ii) grading and sorting of fresh produce [9]; and (iii) inspection of packaging integrity [10]. Real-time detection is commonly achieved with single-shot detectors (e.g., YOLO architectures) [11], while encoder–decoder networks are employed for segmentation tasks [12].

When annotated defect data are scarce, anomaly detection models such as autoencoders or student–teacher networks are applied [13]. Evaluation metrics include precision, recall, F1-score, and mean intersection-over-union (mIoU) for segmentation, which quantify trade-offs between false rejections and missed detections [14].

### 2.3. Spectroscopic and Chemometric Approaches for Composition and Authenticity

Near-infrared (NIR) and hyperspectral imaging (HSI) systems generate high-dimensional spectral datasets [15]. For predictive modeling of compositional traits (e.g., moisture, sugar content, firmness) or authenticity classification, partial least squares regression (PLSR), random forests, gradient boosting machines, and artificial neural networks (ANNs) are widely applied [16,17]. Feature reduction techniques such as principal component analysis (PCA) or genetic algorithms mitigate collinearity [18], while explainable AI tools (e.g., SHAP values) highlight critical wavelengths or metabolites [19].

Model performance is reported using coefficient of determination (R^2^), root mean square error of prediction (RMSEP), and residual predictive deviation (RPD) [20]. In HSI applications, pixel-level prediction maps provide spatial QC information [21].

### 2.4. Sensor-Based and Time-Series Models for Process Monitoring

Inline and environmental signals such as temperature, humidity, vibration, and gas concentrations support predictive QC and process optimization [22]. Tree ensembles, random forests, XGBoost, and neural networks capture nonlinear relationships [23]. Feature engineering improves robustness under small-data conditions [22] and facilitates deployment on embedded systems [24].

Performance metrics include accuracy for fault classification and RMSE, mean absolute error (MAE), and mean absolute percentage error (MAPE) for continuous outcomes [25]. Explainability methods identify controllable process variables for interventions [26].

### 2.5. Packaging and Consumer-Facing Verification

CNN- and YOLO-based frameworks are also widely adopted for packaging QC, including the detection of seal failures and labeling errors [5]. Surrogate models such as support vector regression (SVR) and ANNs predict gas exchange or shelf-life indicators (e.g., CO_2_, ethylene) from measurable correlates [27].

At the consumer interface, mobile recognition systems integrate edge detectors with server-based verification to provide instant allergen or recall information [24]. Key metrics include F1-score, latency per item, and throughput [14].

### 2.6. Formulation Optimization and Nutritional Evaluation

Formulation optimization problems—such as balancing fatty acid profiles, designing growth media for cultivated meat, or maximizing single-cell protein yields—are often solved with ANN regressors combined with metaheuristic search algorithms like genetic algorithms, particle swarm optimization, and NSGA-II [28,29]. Results are reported using R^2^ and RMSE, with Pareto fronts showing trade-offs among nutrition, cost, and sustainability [30].

### 2.7. Evaluation Metrics and Reporting Conventions

Evaluation metrics should be aligned to task type: classification with accuracy, precision, recall, F1, and ROC-AUC; segmentation with mIoU; regression with R^2^, RMSE, RMSEP, and RPD; and forecasting with RMSE, MAE, and MAPE [20]. For industrial deployment, false-reject/false-accept rates, alarm frequency, and throughput are also required [23]. Performance values should be reported in SI units to facilitate comparability [21].

### 2.8. Practical Considerations for Industrial Implementation

Successful deployment requires sensor and lighting standardization [8], calibration and verification protocols [15], human-in-the-loop review for ambiguous cases [7], and integration with manufacturing execution and traceability systems [2]. Model risk management, including drift detection and interpretability, is essential to meet HACCP and ISO frameworks [1].

## 3. Review for QC of Food Industry and ML

Having established this methodological foundation, Section 3 turns from algorithms to applications across product domains. Here, we critically examine how ML techniques are embedded in real-world case studies—including produce, meat, dairy, beverages, packaging, and supply chains—highlighting both the successes and the limitations of their deployment. This transition underscores the central message of the review; ML is not merely a set of abstract tools, but a transformative driver of food quality control, whose practical value depends on careful integration with domain knowledge, real-world constraints, and safety considerations.

### 3.1. Methodology—Literature Search and Selection

**Figure 1:**
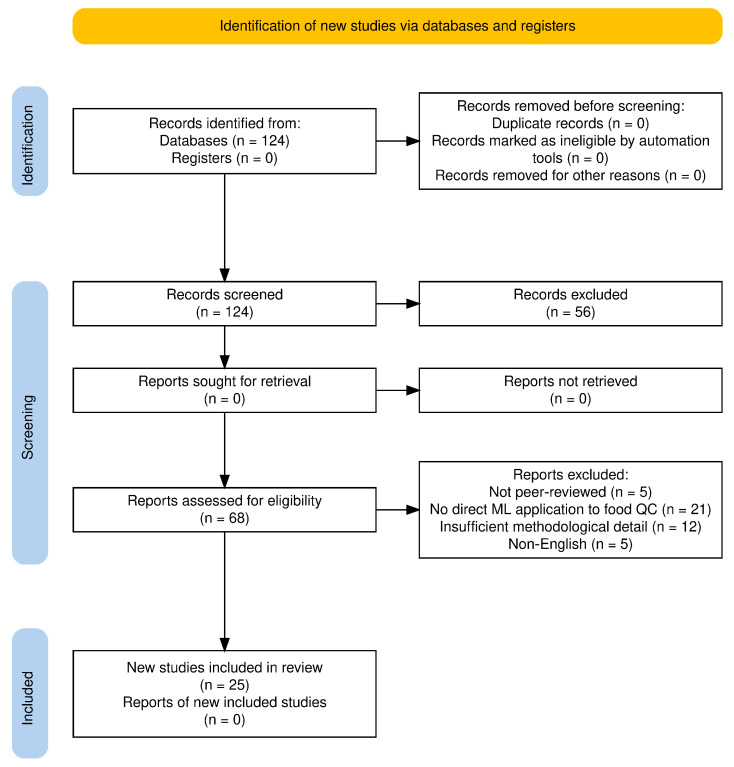
PRISMA 2020 flow diagram of study selection for the systematic review on ML in food quality control.

A structured literature search was conducted in the Scopus database to identify peer-reviewed publications on machine learning applications in food quality control. The search covered the period from January 2005 to March 2025 and used thematic Boolean keyword combinations such as (“machine learning” OR “artificial intelligence”) AND (“food quality control” OR “quality assurance” OR “defect detection” OR “visual inspection” OR “ingredient optimization” OR “predictive analytics” OR “smart packaging” OR “supply chain transparency”). The initial search retrieved 124 records. These were exported from Zotero 7.0.16 (Corporation for Digital Scholarship, Virginia Beach, VA, USA) in RIS format for screening and deduplication. After the removal of duplicates (*n* = 0) and screening of titles and abstracts for relevance, 68 articles remained. Full-text assessment was then performed using the following inclusion criteria: (i) peer-reviewed journal article, (ii) direct application of ML/AI to food quality control, (iii) clear description of methodology and evaluation metrics, and (iv) publication in English. Exclusion criteria were (i) not peer-reviewed (*n* = 5), (ii) no direct ML application to food QC (*n* = 21), (iii) insufficient methodological detail (*n* = 12), and (iv) non-English (*n* = 5). This process resulted in 25 studies meeting all criteria for inclusion in the review. Screening and selection were performed independently by two reviewers, with disagreements resolved by consensus. The literature selection process followed PRISMA 2020 guidelines, and Figure 1 presents the PRISMA flow diagram summarizing the number of records identified, screened, assessed for eligibility, and included, along with reasons for exclusion at each stage [31].

### 3.2. Studies Categorization

Recent advances in ML have enabled a wide range of applications across the food industry, spanning from raw material assessment to end-product inspection, packaging evaluation, and supply chain monitoring. The 25 reviewed studies illustrate the breadth of these applications, which can be grouped by product domain, data modality, and ML methodology (Table 1).

**Figure 2:**
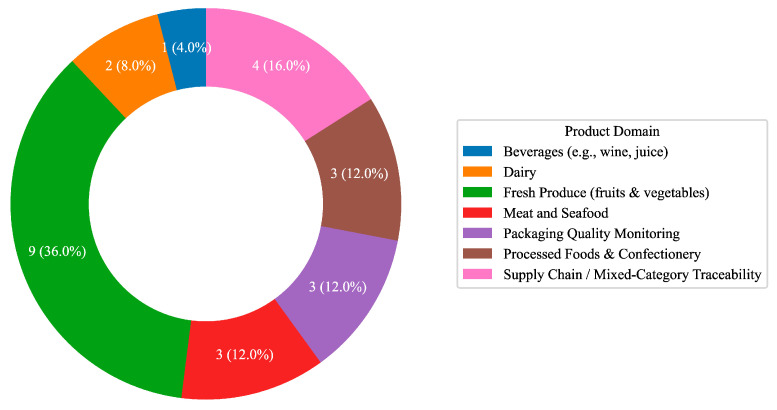
Distribution of reviewed studies by product domain.

From a product domain perspective (Figure 2), the largest share of studies (36%) focused on fresh produce, including hyperspectral imaging for sweet potato quality assessment [19], non-targeted metabolomics for plant-derived materials [32], and predictive modeling of post-harvest gas emissions [33]. Meat and seafood applications (12%) addressed nutritional enhancement [34], cultivated meat culture media optimization [35], and AI-driven drying time prediction [36]. Processed foods (12%) included defect detection in rawhide sticks [37], appearance analysis of 3D-printed chocolate [38], and valorization of food waste into single-cell protein [39]. Packaging QC (12%) featured non-destructive defect detection in reusable containers [14], AI-assisted green packaging design [40], and mobile platforms for consumer-level safety checks [24]. Smaller shares were observed for dairy (8%) [41,42] and beverages (4%) [43], while supply chain/mixed studies (16%) explored vibration-based monitoring [22], traceability systems [44], classification of waste interventions [45], and Industry 4.0 frameworks [46].

**Figure 3:**
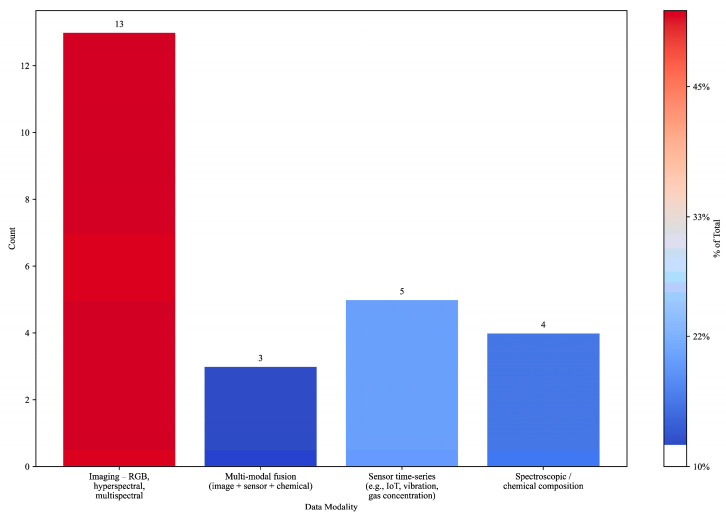
Bar chart illustrating the distribution of data modalities used in food analysis.

In terms of data modality (Figure 3), over half of the studies (52%) relied on imaging—from hyperspectral and RGB image analysis [14,19,24,37,38,47,48] to advanced segmentation models [49]. Spectroscopy/chemical data (16%) supported compositional analysis in wine, olive oil, meat, and fish [34,35,42,43]. Sensor-based approaches (20%) included vibration signal characterization [22], gas emission monitoring [33], and process parameter tracking [50,51]. A smaller subset (12%) integrated multi-modal data, combining imaging, spectral, and sensor inputs for more robust predictions [32,36,44].

**Figure 4:**
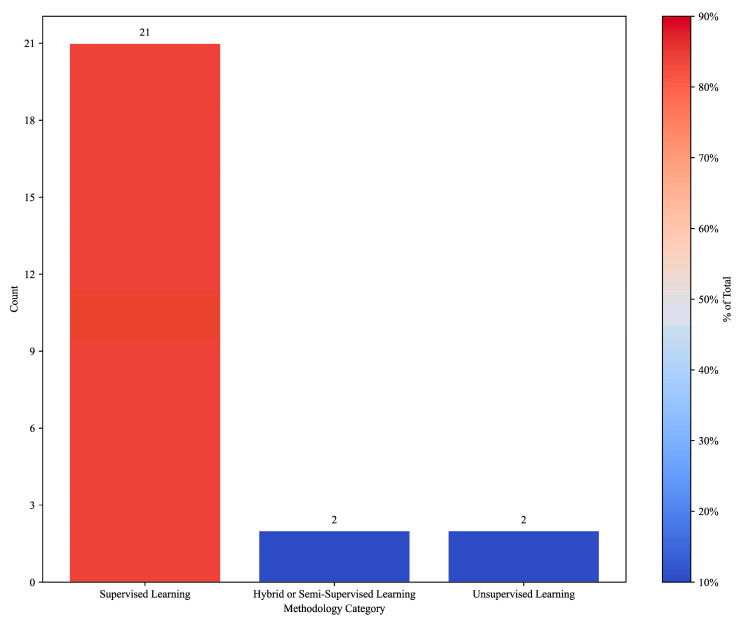
ML methodology distribution by product domain.

Regarding ML methodology (Figure 4), supervised learning dominated (84%), with models such as convolutional neural networks, random forests, and gradient boosting applied to classification, regression, and prediction tasks [19,22,24,32,33,34,35,36,38,39,40,41,42,43,45,46,47,48,51,52]. Unsupervised learning (8%) was used for anomaly detection and clustering in defect identification [14,37], while hybrid approaches (8%) combined supervised and unsupervised techniques, as in open-world ingredient segmentation [49] and energy optimization under Industry 5.0 [50].

Collectively, these studies demonstrate that ML is no longer confined to niche applications in food QC. Instead, it is becoming a core enabler of scalable, precise, and responsive quality management—addressing the limitations of traditional, reactive QC methods and meeting the demands of globalized supply chains and evolving consumer expectations.

In addition to the 25 systematically reviewed studies presented in Table 1, it is worth highlighting emerging applications of machine learning in green extraction technologies. For example, Mantiniotou et al. [53] applied ensemble ML models—including Random Forest, Gradient Boosting, and AdaBoost—to optimize the pressurized liquid extraction (PLE) of rosemary for antioxidant recovery. Their approach combined experimental modeling with synthetic data augmentation to enhance prediction accuracy and process efficiency. This study exemplifies how AI-assisted extraction workflows can reduce reliance on labor-intensive experimentation and support the sustainable development of bioactive compounds for food and nutraceutical applications.

**Table 1:** Classification of the 25 reviewed studies by food type, data nature, and applied ML algorithms.

Category Axis	Sub-Category	Count	% of Total	Representative Studies
Product Domain	Fresh produce	9	36	[19,32,33,44]
	Dairy	2	8	[41,42]
	Meat and seafood	3	12	[34,35,36]
	Beverages	1	4	[43]
	Processed foods	3	12	[37,38,39]
	Packaging QC	3	12	[14,24,40]
	Supply chain/mixed	4	16	[22,45,46]
Data Modality	Imaging	13	52	[14,19,24,37,38,47,48,49]
	Spectroscopy/chemical	4	16	[34,35,42,43]
	Sensors	5	20	[22,33,41,50,51]
	Multi-modal	3	12	[32,36,44]
ML Methodology	Supervised	21	84	[19,22,24,32,33,34,35,36,38,39,40,41,42,43,45,46,47,48,51,52]
	Unsupervised	2	8	[14,37]
	Hybrid	2	8	[49,50]

### 3.3. Food Quality Applications

This section documents the use of ML in compositional analysis, grading, and authenticity assessment across various food product categories. Here, studies detail how feature extraction from spectral, imaging, and sensor data, in concert with advanced classifiers, is enabling more precise and granular food quality characterization.

Ahmed et al. [19] presented a non-destructive quality assessment approach for sweet potatoes using Visible and Near-Infrared Hyperspectral Imaging (VNIR-HSI) combined with Explainable Artificial Intelligence (XAI). The study targeted three key attributes: dry matter content (DMC), soluble solid content (SSC), and firmness, using samples from three varieties. Spectral data (400–1000 nm) were pre-processed, and key wavelengths were selected via Genetic Algorithm (GA) and Competitive Adaptive Reweighted Sampling (CARS). Predictive modeling employed supervised PLSR, with interpretability achieved through SHapley Additive exPlanations (SHAP). The method achieved high accuracy for DMC, good performance for firmness, and moderate performance for SSC. Prediction maps enabled pixel-level visualization of quality attributes, supporting efficient grading in the food industry. In the same application, Ahmed et al. [19] demonstrated that coupling hyperspectral imaging with XAI could achieve over 95% classification accuracy for anthocyanin content in sweet potatoes, while generating feature-importance maps aligned with known pigment absorption bands. This highlights the potential of integrating spectral data with XAI to meet both performance and transparency requirements in fresh-produce QC.

Pan et al. [32] developed a two-stage analytical framework combining non-targeted metabolomics with explainable AI for QC of Ginger-Eucommiae Cortex (G-EC). In Stage 1, non-targeted ultra-performance liquid chromatography–high-resolution mass spectrometry (UPLC-HRMS) with multivariate statistical analysis identified 517 significantly altered metabolites between raw and optimally processed G-EC, with coniferyl aldehyde (CFA) emerging as a key quality marker. In Stage 2, a supervised Extreme Gradient Boosting (XGBoost) regressor was trained on color features, extracted via watershed-based image segmentation to predict CFA content. SHAP analysis ranked the most influential color variables, revealing that a*, L, and E values had the highest impact on CFA prediction. The model achieved high predictive performance with no evidence of overfitting, demonstrating the feasibility of rapid, non-destructive CFA quantification for food and medicinal product QC.

Ding et al. [37] developed a data augmentation framework to address limited training samples in defect detection for rawhide stick products, a pet food item with irregular shapes. The proposed model integrates a Residual Block (ResB) and Hybrid Attention Mechanism (HAM) into a Deep Convolutional Generative Adversarial Network (DCGAN) architecture, termed ResB–HAM–DCGAN, and employs a Wasserstein loss with gradient penalty to stabilize training and improve image fidelity. The augmented dataset was used to train DL models for defect classification, with defects including surface stains and irregular shapes. The model generated high-quality synthetic images from an original dataset of 1800 grayscale images. Performance was evaluated against standard DCGAN and Wasserstein GAN with Gradient Penalty (WGAN-GP) using Inception Score (IS), Fréchet Inception Distance (FID), and Structural Similarity Index Measure (SSIM). ResB–HAM–DCGAN outperformed baselines. When used to augment the dataset for training a LeNet-5 CNN, the approach improved classification accuracy and reduced loss compared to other augmentation methods.

Jauhar et al. [41] proposed a smart sensing system for real-time monitoring of milk spoilage using Internet of Things (IoT) sensors and ML. The system integrates temperature, pH, and gas concentration sensors to collect spoilage-related data, which is transmitted wirelessly to a processing unit. Data preprocessing included noise filtering and normalization, followed by supervised model training for spoilage classification. The authors evaluated multiple algorithms, including RF, SVM, and KNN, to identify the optimal model. The RF model achieved the highest accuracy and lowest error rate, outperforming SVM and KNN. The system demonstrated potential for continuous, non-invasive quality monitoring in the dairy supply chain, enabling early spoilage detection and reducing waste. In the same work, Jauhar et al. [41] employed explainable AI to enhance the resilience of perishable-product supply chains by leveraging customer-profile data, enabling targeted interventions to reduce spoilage and optimize distribution routes.

Kurtanjek [42] applied causal AI modeling to integrate physicochemical data and consumer sensory assessments for food quality analysis. The study used three datasets: wheat baking quality, 45 physicochemical variables from 7 cultivars over 3 years; fermented dairy product quality, 1059 samples with pH, temperature, taste, odor, fat, turbidity, and color; and wine quality, with 1599 red and 4898 white samples, with 12 composition variables. For feature selection, the wheat dataset was regularized using a Least Absolute Shrinkage and Selection Operator (LASSO) elastic net, reducing it to 10 key variables. A supervised RF regression model achieved 75% variance explanation in cross-validation. Structural Causal Models (SCM) were constructed using Bayesian networks (BN) with Monte Carlo Markov Chain (MCMC) sampling to infer directed acyclic graphs (DAG) and average causal effects (ACE), validated by Double Machine Learning (DML). In the dairy dataset, a supervised RF classifier achieved <1% out-of-bag classification error. Causal analysis identified temperature and fat content as primary direct causes. In the wine dataset, RF regression models achieved prediction errors of 5.13% for red and 4.17% for white wine. Alcohol content showed the highest positive ACE = 0.35 quality/alcohol unit, while volatile acidity had the highest negative ACE = −0.2 quality/acidity unit.

A detailed summary of the reviewed works, including data size, modeling methods, and evaluation metrics, is presented in Table 2.

#### Overview of Food Quality Applications

**Figure 5:**
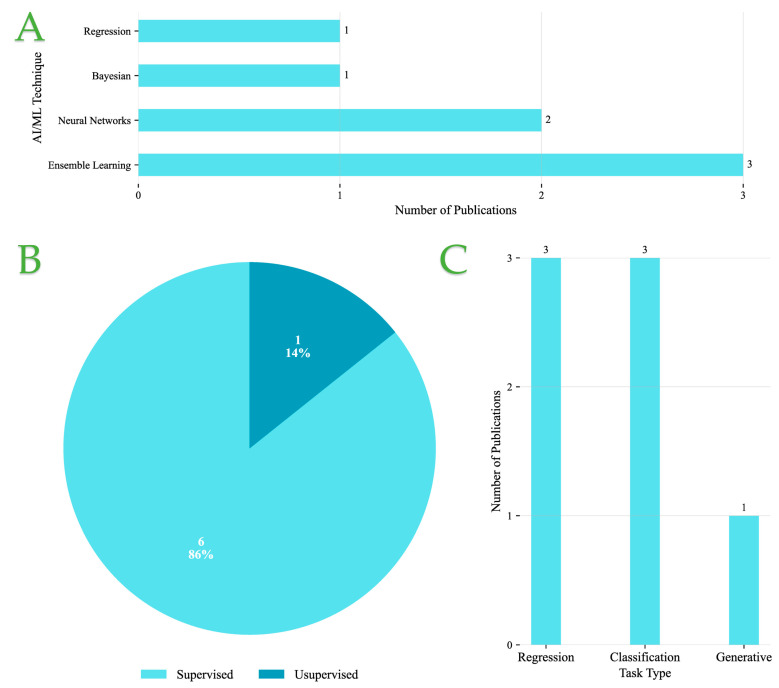
(**A**) Distribution of AI/ML techniques in Food Quality Applications category. (**B**) Types of learning approaches used in Food Quality Applications category. (**C**) Prediction task in Food Quality Applications category.

Figure 5A presents the distribution of AI/ML techniques employed across the reviewed publications. Ensemble learning and NN-based approaches are the most frequently adopted, appearing in three and two studies, respectively, highlighting their strong ability to capture complex feature interactions. By contrast, Bayesian methods and regression-based models are less represented, with only one study each, indicating their use in more specialized or constrained contexts.

Figure 5B shows that supervised learning overwhelmingly dominates, accounting for 86% of the studies. Unsupervised methods are rarely used, with only a single instance (14%), suggesting that while exploratory approaches have potential, most applications still rely on labeled data for predictive accuracy.

Finally, Figure 5C illustrates the categorization of tasks. Regression and classification each appear in three studies, reflecting their central role in predictive modeling tasks. In contrast, generative modeling is seldom applied, with only one instance, indicating limited exploration of data synthesis or augmentation approaches.

Overall, these findings point to a methodological preference for supervised paradigms combined with ensemble and NN techniques, primarily applied to regression and classification problems. This trend emphasizes the community’s focus on leveraging well-established predictive models, while exploratory methods such as unsupervised and generative learning remain underutilized.

### 3.4. Defect Detection and Visual Inspection Systems

The second thematic cluster delves into ML-powered defect and contamination identification. Emphasis is placed on computer vision-based inspection systems—especially CNN-driven models for detecting bruises, discolorations, extraneous matter, and subtle packaging flaws—in fruits, vegetables, meats, grains, and packaged foods.

Huang et al. [38] investigated the application of AI-based image recognition for QC in three-dimensional (3D) printed chocolate incorporating different oleogels, namely monoglycerides (MAG), sucrose fatty acid ester (SE), and hydroxypropyl methylcellulose (HPMC). The study examined the effects of these oleogels on the thermal and textural properties of white and dark chocolate, as well as their ability to maintain printed structural integrity. Initially, an AI-driven image recognition model, implemented as a supervised ANN, was trained on images of chocolates with varying shapes and formulations. The system extracted geometric and contour-based features to identify deviations from the intended designs, achieving recognition rates exceeding 90% for circular shapes and between 70% and 85% for square or triangular forms. Subsequently, the trained ANN was deployed for defect detection in a simulated production line environment, where chocolates were moved to emulate a conveyor belt inspection process. This stage achieved high accuracy (>90%) in detecting major structural defects such as incomplete extrusion of more than three layers, although performance declined to below 50% for subtle imperfections. The findings demonstrate the potential of AI-based image recognition as a non-invasive tool for real-time quality monitoring in additive manufacturing of confectionery products, while also highlighting the need for enhanced detection capabilities for minor defects.

Truong and Luong [14] proposed a non-destructive anomaly detection framework for reusable food packaging using a student–teacher autoencoder architecture with vision transformers. The dataset comprised 245 high-resolution RGB images from four cup categories. Initially, a background removal algorithm isolated the cup region. The anomaly detection model employed Data-Efficient Image Transformer (DeiT) backbones for both student and teacher networks, with the teacher pretrained on ImageNet and the student trained solely on defect-free images to replicate the teacher’s feature outputs. Defective samples were identified through feature discrepancies between the two networks, using cosine-distance-based hard-feature loss to focus training on challenging areas. Performance was enhanced through data augmentation like rotation, brightness-contrast adjustments, as well as corruption models like stained-shape and latent noise, and knowledge transfer from other cup types using diffusion-based synthetic defect generation. The best configuration diffusion-based transfer, combined with brightness-contrast adjustment and rotation, achieved an image-level F1-score of 0.969, with pixel-level F1-scores of 0.508. The method proved effective even with minimal training data and showed potential for extension to defect detection in other mass-produced food-related products. In the same work, Truong and Luong [14] developed a comparable non-destructive, autoencoder-based defect-detection system for reusable food packaging, achieving high sensitivity in identifying contamination and structural flaws, thereby reinforcing circular-economy objectives in packaging systems.

Aiello and Tosi [43] evaluated three supervised ML classifiers—RF, Linear Discriminant Analysis (LDA), and KNN for predicting wine quality, vineyard origin, and olive oil geographical origin from chemical composition data. Three datasets were used: (1) a dataset containing 6800 Portuguese red and white wine samples with 11 physicochemical variables and quality scores; (2) a dataset of 178 Italian wine samples from 3 vineyards with 13 chemical characteristics; and (3) a dataset of 572 olive oil samples from 9 Italian regions with seven fatty acid measurements. Data were preprocessed with the Synthetic Minority Over-sampling Technique (SMOTE) to address class imbalance. Across all tasks, RF achieved the best performance. For wine quality prediction, RF attained accuracies of 0.63 for white and 0.65 red with average ROC–AUC of 0.86 and 0.77. For vineyard origin classification, RF achieved perfect accuracy and ROC–AUC of 1.00. For olive oil origin classification, RF reached 0.96 accuracy and 1.00 ROC–AUC. Feature importance analysis identified free sulfur dioxide, chlorides, and alcohol as top predictors for white wine; alcohol, volatile acidity, and sulfates for red wine; flavonoids, color intensity, and proline for vineyard origin; and linolenic, linoleic, and eicosenoic acids for olive oil origin. In the same work, Aiello and Tosi [43] developed an AI-driven tool for predicting “unhealthy” wine and olive oil batches using chemical profiling and supervised learning, demonstrating the applicability of such models to beverage-quality control for early detection of off-spec products.

Cengel et al. [47] developed a DL-based system for the automatic detection of egg surface damage to improve QC in the food industry. The dataset contained 794 color images, 632 damaged and 162 intact, captured under diverse real-world conditions and was split into 80% training and 20% testing sets. Four supervised CNN classifiers—GoogLeNet, Visual Geometry Group (VGG)-19, MobileNet-v2, and Residual Network (ResNet)-50—were trained and compared. Among the models, GoogLeNet achieved the highest classification performance with an accuracy of 98.73%, precision of 98.41%, recall of 100%, and F1-score of 99.2%, although it had the longest training time, 556 s. MobileNet-v2 trained fastest in 443 s, but achieved slightly lower precision. VGG-19 had perfect precision 100% but slightly lower recall than GoogLeNet, while ResNet-50 recorded the lowest accuracy 96.84%. The study demonstrated that deep CNN architectures can provide highly accurate, efficient, and non-invasive solutions for real-time egg damage detection, reducing product losses and improving safety in food supply chains.

Chen et al. [48] developed an intelligent defect detection system for gummy candies operating within an edge computing and AI of Things (AIoT) framework. The system aimed to replace manual visual inspection, reducing labor requirements and improving production efficiency. The dataset consisted of 5000 manually captured images of defective gummy candies across four defect categories, hole, leakage, abnormal color, and connection augmented to 20,000 images through flipping, saturation adjustment, and contrast modification. Initially, images were acquired in real time using a charge-coupled device (CCD) camera integrated into a conveyor-belt inspection setup. The captured images were processed by a YOLOv3 (You Only Look Once) CNN for object detection. The model architecture used Darknet-53 as a backbone with multi-scale feature fusion to detect large, medium, and small defects in a single forward pass. Logistic regression classifiers with binary cross-entropy loss were employed for multi-label defect prediction. The trained system achieved a precision of 93%, recall of 87%, and F1-score of 90%, with an average detection speed of 3.2 items per second. The results demonstrated the feasibility of integrating YOLO-based defect detection into edge computing architectures for real-time QC in the confectionery industry, though limitations remained in detecting certain three-dimensional surface defects.

A detailed summary of the reviewed works, including data size, modeling methods, and evaluation metrics, is presented in Table 3.

#### Overview of Defect Detection and Visual Inspection Systems

**Figure 6:**
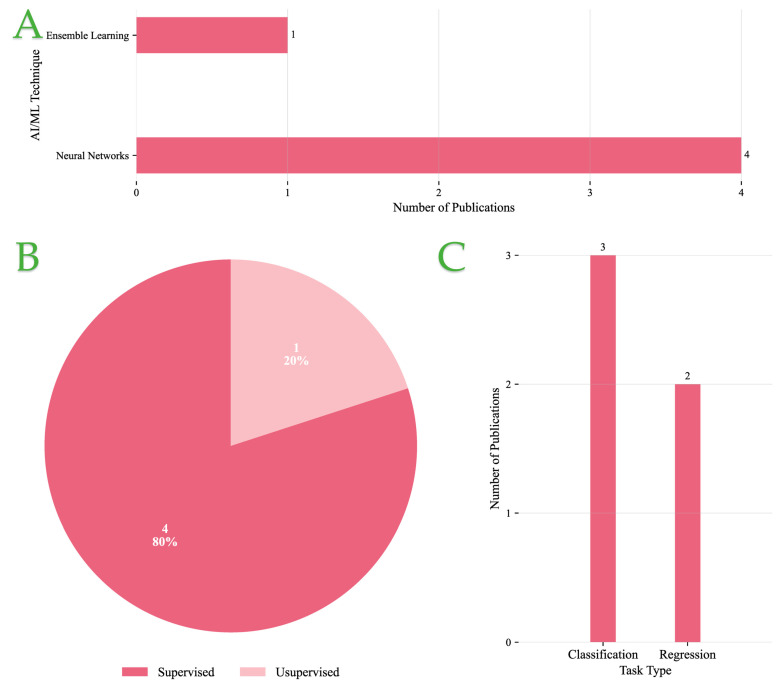
(**A**) Distribution of AI/ML techniques in Defect Detection and Visual Inspection Systems category. (**B**) Types of learning approaches used in Defect Detection and Visual Inspection Systems category. (**C**) Prediction task in Defect Detection and Visual Inspection Systems category.

Figure 6A presents the distribution of AI/ML techniques applied within Defect Detection and Visual Inspection Systems. NN-based approaches are clearly dominant, featured in four studies, underscoring their robustness in handling image-based pattern recognition and classification tasks. Ensemble learning, by contrast, appears in only one study, suggesting that its application remains relatively limited in this context.

Regarding learning paradigms, Figure 6B reveals a strong prevalence of supervised learning methods, accounting for 80% of the reviewed studies. Only a single instance (20%) employed unsupervised learning, indicating that most research continues to rely on labeled datasets and explicitly defined targets for training models.

Figure 6C illustrates a slight preference for classification over regression. Classification tasks—used to determine categorical outputs such as defect type—appear in three studies, while regression, applied to estimate continuous variables like defect severity, is employed in two studies. This near parity reflects the dual needs of both categorical defect identification and quantification of defect-related metrics.

Overall, the data point to a methodological inclination toward supervised NN-based models, with both classification and regression playing complementary roles in the formulation of defect detection tasks.

### 3.5. Ingredient Optimization and Nutritional Assessment

This section focuses on ML-driven ingredient formulation, predictive modeling for nutritional composition, and dynamic optimization of additive or substitution scenarios. ML-based platforms for virtual formulation, such as Bayesian optimization and ensemble regressors, are evaluated alongside their impact on sustainability and product performance.

Sadhu et al. [34] developed a hybrid AI approach to optimize the frying conditions of Catla in mustard oil, aiming to maximize nutritional quality while minimizing energy and resource waste. Fresh filets, 10 kg in total, were fried under systematically varied temperatures from 140 to 240 °C, for 5–20 min, and oil amounts from 25 to 100 mL/kg per fish, yielding 28 experimental runs, each in triplicate. Nutritional quality was assessed via the polyunsaturated-to-saturated fatty acid ratio (PUFA/SFA) and the index of atherogenicity (IA). A supervised ANN with a multi-layer perceptron architecture was trained to model the nonlinear relationship between frying parameters and nutritional indices, achieving particularly strong predictive performance for IA. This ANN was then coupled with metaheuristic optimizers—genetic algorithm, particle swarm optimization (PSO), and multi-objective genetic algorithm (MOGA)—to determine optimal frying conditions. Single-objective optimization improved PUFA/SFA by up to 63.05% and reduced IA by up to 99.64% compared to baseline frying, while MOGA achieved simultaneous improvements of 44.76% in PUFA/SFA and 92.94% in IA at 118.92 °C, 6.06 min, and 40 mL oil/kg. Validation experiments confirmed predictive accuracy, with absolute relative errors under 5% for PUFA/SFA and under 10% for IA. In the same work, Sadhu et al. [34] applied a comparable AI-driven optimization framework to enhance the nutritional value of fried fish, demonstrating how such methodologies could be adapted to dairy processing to optimize nutrient retention and sensory quality under varying thermal treatments.

Nikkhah et al. [35] proposed an AI–based multi-objective optimization framework to formulate a reduced-serum culture medium for cultivated zebrafish meat, integrating environmental, economic, and biological performance metrics. The dataset consisted of 93 experimental formulations, each tested in triplicate, designed via Response Surface Methodology (RSM) using seven independent variables—insulin-like growth factor, fibroblast growth factor, transforming growth factor, platelet-derived growth factor, selenium, ascorbic acid, and fetal bovine serum. Dependent variables were global warming potential (GWP), ingredient cost, and cell-specific growth rate. Radial Basis Function (RBF) NNs were employed to model the three dependent variables, achieving high predictive accuracy. These models served as inputs to a Non-dominated Sorting Genetic Algorithm II (NSGA-II), which identified optimal formulations that maximized growth rate while minimizing GWP and cost. Validation experiments confirmed negligible deviation from predicted values. Compared to average formulations, the optimized medium reduced GWP by up to 65%, decreased cost by up to 24%, and increased growth rate by up to 51%. This demonstrates the feasibility of AI-driven serum-reduction strategies for sustainable fish cell culture media in cultivated meat production.

Sagar et al. [39] investigated the valorization of food waste into single-cell protein (SCP) using Pichia occidentalis PG5, combining statistical optimization and AI modeling. The study used salad peel waste and leftover food waste hydrolysates as substrates, assessing multiple process variables through one-factor-at-a-time (OFAT), Plackett–Burman design, and Central Composite Design (CCD). Four significant parameters—salad peel waste hydrolysate concentration, malt extract concentration, calcium chloride concentration, and pH—were identified for optimization. For predictive modeling, a supervised SVM regressor with a linear kernel was developed in R and compared to the Response Surface Methodology (RSM) model. The SVM model demonstrated superior predictive performance with R^2^ = 0.9772 compared to RSM with R^2^ = 0.8881. Under optimal SVM-predicted conditions like, 50 g/L salad peel waste hydrolysate, 20 g/L malt extract, 20 g/L CaCl_2_, pH 8.0, experimental SCP yield reached 25.90 g/L, representing a ~16-fold increase compared to unoptimized conditions.

Chen et al. [49] introduced Ingredient Segment Anything Model (IngredSAM), a one-shot, open-world food ingredient semantic segmentation framework that requires no model training. Initially, a multi-level visual feature extraction was performed using four visual foundation models—DINOv2, Masked Autoencoder (MAE), CLIP, and I-JEPA—to obtain semantically consistent representations from both a clean ingredient prompt image and an open-world food image. These features were aggregated and processed using the unsupervised Texture-guided Saliency Distilling Network (TSDN) to isolate the ingredient’s foreground in the prompt image. Afterwards, cosine similarity between prompt and open-world image features was used to generate positive and negative point prompts, which guided the IngredSAM to produce the final segmentation mask. The method was evaluated on FoodSeg103 and UECFoodPix Complete datasets. IngredSAM achieved the highest mIoU on both datasets, outperforming supervised and open-world baselines. On FoodSeg103, it reached an mIoU of 48.78%, with the top ingredient being bread, with IoUs of 69.17%, and 67.23% for carrot. On the UECFoodPix Complete dataset, it achieved 70.21% mIoU, with the best category of rice reaching 76.47% IoU. The results demonstrated strong generalization to diverse ingredient appearances and robustness in complex, unconstrained scenes.

A detailed summary of the reviewed works, including data size, modeling methods, and evaluation metrics, is presented in Table 4.

#### Overview of Ingredient Optimization and Nutritional Assessment

**Figure 7:**
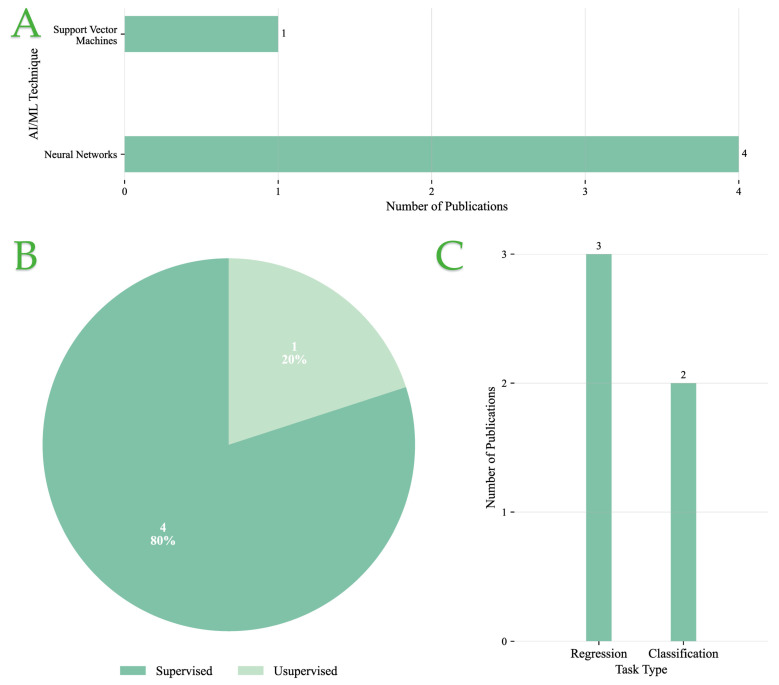
(**A**) Distribution of AI/ML techniques in Ingredient Optimization and Nutritional Assessment category. (**B**) Types of learning approaches used in Ingredient Optimization and Nutritional Assessment category. (**C**) Prediction task in Ingredient Optimization and Nutritional Assessment category.

Figure 7A illustrates the distribution of AI/ML methods applied to Ingredient Optimization and Nutritional Assessment tasks. NNs are the predominant technique, appearing in four studies, reflecting their high adaptability and effectiveness in modeling complex, nonlinear relationships in nutritional datasets. SVMs are reported in only one study, indicating a comparatively limited use in this application area.

As shown in Figure 7B supervised learning dominates the field, comprising 80% of the studies. This strong preference suggests that ingredient optimization and nutritional analysis tasks are primarily approached using labeled datasets, where target outputs such as nutritional values or ingredient combinations are known. Only a single study (20%) utilized unsupervised learning, pointing to minimal exploration of data-driven discovery without explicit labels in this domain.

Regarding task formulation, Figure 7C reveals a slight leaning toward regression tasks (three studies), which are typically used to predict continuous nutritional values or optimize ingredient ratios. Classification tasks, such as categorizing products based on nutritional quality or dietary constraints, were reported in two studies. This balanced representation underscores the dual objectives of precise nutrient estimation and categorical decision-making in nutritional assessment systems.

In summary, supervised NN-based models are the preferred choice for Ingredient Optimization and Nutritional Assessment, with regression tasks being slightly more prevalent than classification, reflecting the continuous nature of nutritional variables often encountered in such applications.

### 3.6. Packaging—Sensors and Predictive QC

Here, the review explores ML’s role in predicting packaging failures, optimizing design, and automating inspection for defects, seal integrity, and label compliance. DL and sensor fusion systems are shown to be essential for high-speed, non-destructive, and reliable packaging QC on production lines.

Rashvand et al. [33] investigated the effect of dielectric barrier discharge (DBD) cold plasma combined with modified atmosphere packaging (MAP) on the postharvest quality and shelf life of ‘Shahroudi’ apricots stored at 21 °C for 12 days. Physicochemical parameters included mass loss, pH, soluble solids content, titratable acidity, and skin color. Mechanical properties included Young’s modulus, tangent modulus, and bioyield stress. In-package gas composition, O_2_, CO_2_, and ethylene production were monitored. Additionally, bruise susceptibility was evaluated using pendulum impact tests and scanning electron microscopy (SEM). Data from mass loss, pH, soluble solids content, titratable acidity, skin color, and bioyield stress were used as inputs to two ML models—an ANN and an SVR model—to predict CO_2_ and ethylene production. The optimal ANN architecture was an MLP with two hidden layers of 17 neurons each for CO_2_ prediction (R^2^ = 0.983, RMSE = 0.476) and 15 neurons each for ethylene prediction (R^2^ = 0.933, RMSE = 5.376). The best SVR performance was achieved using a radial basis function (RBF) kernel for CO_2_ (R^2^ = 0.894, RMSE = 6.077) and ethylene (R^2^ = 0.759, RMSE = 14.117), outperforming polynomial kernels. ANN consistently outperformed SVR in predictive accuracy for both gases. The study concluded that MAP and DBD treatments, particularly at 10–15 min, improved the retention of quality attributes and reduced CO_2_ and ethylene production compared to MAP alone or untreated controls. The proposed ANN model provided robust predictions, demonstrating the potential for integrating intelligent modeling into postharvest packaging digitalization strategies.

Dai [40] developed an AI–based methodology for designing green and low-carbon food packaging, integrating wireless sensor networks (WSN) with ANN to optimize energy use, minimize pollution, and improve packaging efficiency. The framework utilized an assurance weight information selection method to classify intelligent energy-saving packaging types, with ANN forming an energy consumption vector from WSN-acquired data. Packaging design parameters were evaluated for their impact on environmental footprint across the full life cycle, from material selection to manufacturing, transportation, and disposal. The proposed AI-WSN model was compared against three baseline approaches: traditional ML, Cognitive Big Data Analysis (CBDA), and IoT–based methods. Across metrics, AI-WSN achieved the lowest pollution rate, 70% vs. 90–91% for others; lowest energy consumption rate, 69% vs. 78–92%; and fastest computation time, 20 units vs. 43–60. The model also reached a classification accuracy of 97.6% for intelligent packaging type determination. These results highlight the effectiveness of combining WSN data acquisition with ANN-based optimization for sustainable packaging design.

Park et al. [24] developed a mobile food safety inquiry platform capable of real-time packaged food recognition and safety verification using DL. The system integrates a smartphone-based application with an AI server, enabling users to capture product images and instantly retrieve safety data, including ingredient lists, nutritional facts, and recall status. The core model was a fine-tuned YOLOv7-E6E architecture trained on a custom dataset of 80,000 images from 100 imported food and beverage types, collected under varied lighting, angles, and framing conditions to ensure robustness. The YOLOv7-E6E detector demonstrated high performance, achieving precision = 99.23%; recall = 100%; F1-score = 99.46%, on the test set (4000 images). Real-world smartphone testing yielded 98% accuracy across 2000 trials, even with challenging capture conditions. Usability tests with 71 participants confirmed significant efficiency gains over QR code or internet-based methods, reducing task times by ~70% and improving recall-check completion rates from 68% to 96%.

Luque et al. [22] presented a fault detection framework for health monitoring of gripping pliers in beverage bottling plants, a critical agri-food manufacturing process. Thirteen experiments were conducted under three health states, healthy, spring-damaged, and bearing-damaged, producing 497 vibration signal segments of 5 s each, recorded via single-axis accelerometers at 12.8 kHz. Three feature extraction strategies were compared: raw features with 64,000 values, specialized kurtosis- and RMS-based features with 2 values, and a 33-variable set of generic time and frequency domain features. A supervised RF classifier was trained to distinguish health states. Generic features achieved the best performance, with 100% classification accuracy on the testing set, outperforming specialized features (88% accuracy) and raw features (83% accuracy). The generic features also demonstrated superior robustness to Gaussian noise, requiring fewer training samples and exhibiting lower computational cost relative to accuracy. The results confirmed that generic feature-based RF classification is an efficient and scalable method for predictive maintenance in industrial food processing equipment.

A detailed summary of the reviewed works, including data size, modeling methods, and evaluation metrics, is presented in Table 5.

#### Overview of Packaging—Sensors and Predictive QC Category

**Figure 8:**
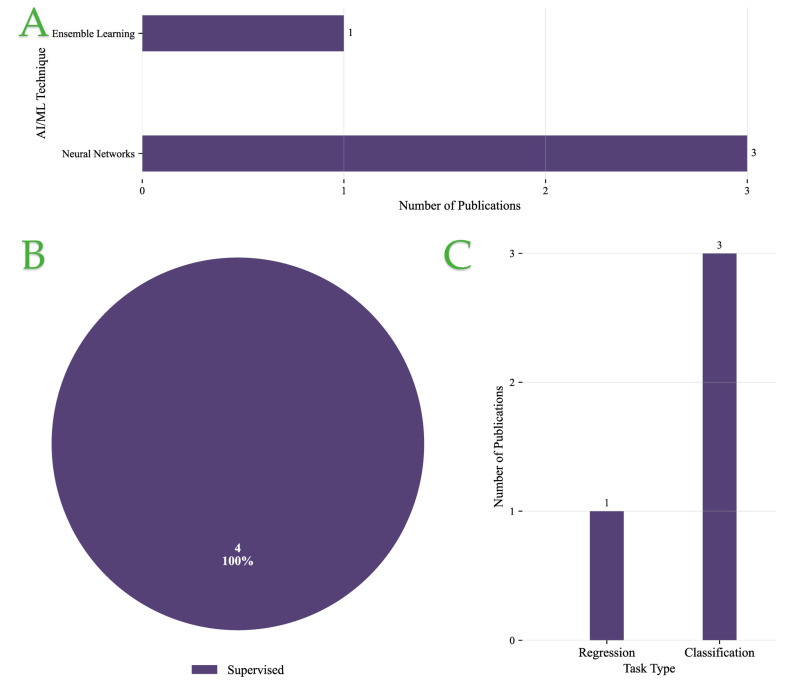
(**A**) Distribution of AI/ML techniques in Packaging—Sensors and Predictive QC category. (**B**) Types of learning approaches used in Packaging—Sensors and Predictive QC category. (**C**) Prediction task in Packaging—Sensors and Predictive QC category.

Figure 8A presents the distribution of AI/ML techniques applied in the packaging, sensors, and predictive QC category. NNs are the most widely utilized, appearing in three studies, underscoring their adaptability in capturing complex sensor signals and packaging-related data patterns. Ensemble learning is less represented, with only one study, suggesting its use is still at the exploratory stage in this domain.

As shown in Figure 8B, supervised learning accounts for 100% of the reviewed publications, reflecting the field’s complete reliance on labeled data for predictive modeling. No unsupervised approaches have yet been reported, indicating that methods for discovering hidden structures in packaging or sensor data remain unexplored.

Regarding task type, Figure 8C shows that classification dominates, with three studies, while regression is applied in only one study. This distribution suggests that predictive QC and packaging-related AI tasks are primarily framed as categorical decisions, such as defect detection or product classification, rather than continuous prediction.

Overall, these results highlight a strong methodological preference for supervised NN–based approaches in packaging, sensors, and predictive QC applications, with classification tasks forming the core research focus.

### 3.7. Supply Chain—Traceability and Transparency

This domain investigates how ML, often integrated with blockchain and IoT, is transforming traceability, provenance verification, and fraud/adulteration detection across complex supply chains. Case studies highlight frameworks for data-driven transparency, rapid recall initiation, and regulatory compliance.

Hassoun et al. [44] reviewed the application of fourth-industrial-revolution technologies, termed Traceability 4.0, in fruit and vegetable supply chains to enhance authenticity, safety, and quality. The study synthesized evidence from multiple case applications involving AI, the IoT, blockchain, and Big Data (BD), contrasting these with conventional traceability tools such as chromatographic, spectroscopic, isotopic, and biomolecular methods. AI implementations included computer vision systems for automated grading of blueberries and apples, ML–based quality assessment of kiwifruit and carrots, and NN models for defect detection in dried fruits and vegetables. IoT deployments utilized sensor networks, QR and RFID codes for real-time monitoring of environmental parameters like humidity, temperature, CO_2_, and product location from farm to consumer. Blockchain trials integrated IoT data to create immutable transaction records, improving transparency, query efficiency, and stakeholder trust, while BD analytics supported predictive modeling for supply chain efficiency, waste reduction, and resource optimization. Reported outcomes included up to 20% yield improvements in field monitoring systems, 98% classification accuracy in origin authentication of avocados via isotopic and elemental analysis integrated with AI, and enhanced fraud prevention through combined blockchain–AI systems. Despite these gains, large-scale adoption remains constrained by high implementation costs, limited interoperability standards, and infrastructure and skills gaps. The authors conclude that integrated AI–IoT–blockchain–BD systems, supported by interdisciplinary collaboration and standardization, are required for scalable, industrial-grade Traceability 4.0 in the horticultural sector.

Zou et al. [45] developed two supervised NN models to classify food waste interventions across the global food supply chain using natural language processing (NLP). A dataset of 2469 interventions was compiled from 154 scholarly articles published between 2013 and 2023, with 478 examples manually classified into six intervention types and seven stakeholder groups to form the training set. Model 1 performed multi-label classification of stakeholder groups using a CNN with GloVe embeddings, while Model 2 performed multi-class classification of intervention types using the Universal Sentence Encoder (USE) and a fully connected NN. Performance evaluation revealed that Model 1 achieved the highest scores overall, with an F1-score of 0.96, outperforming Model 2 with F1-score of 0.90. The results demonstrate that supervised CNN-based text classification is an effective and scalable method for systematically organizing food waste interventions, enabling faster meta-analysis and informed decision-making for stakeholders.

A detailed summary of the reviewed works, including data size, modeling methods, and evaluation metrics, is presented in Table 6.

#### Overview of Supply Chain—Traceability and Transparency Category

**Figure 9:**
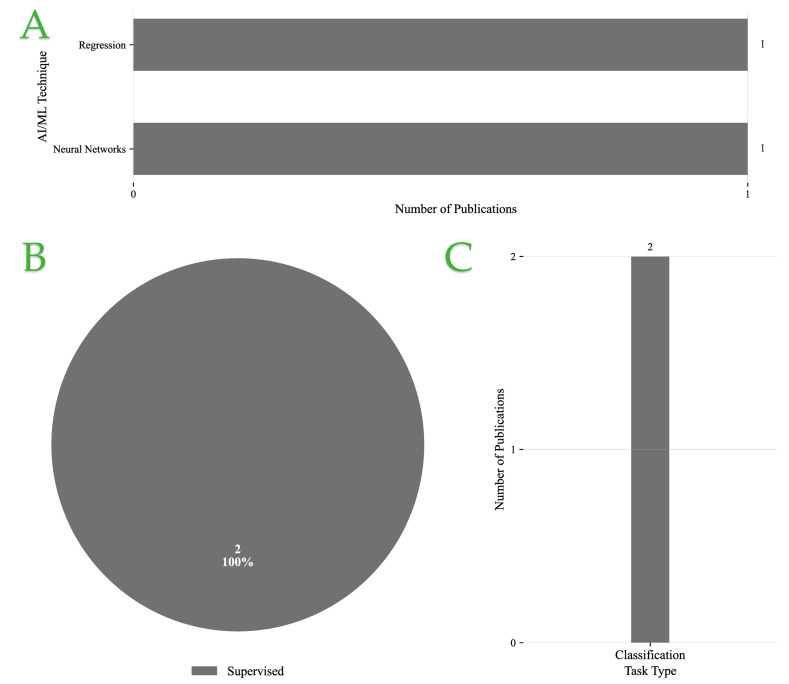
(**A**) Distribution of AI/ML techniques in Supply Chain—Traceability and Transparency category. (**B**) Types of learning approaches used in Supply Chain—Traceability and Transparency category. (**C**) Prediction task in Supply Chain—Traceability and Transparency category.

Figure 9A shows the distribution of AI/ML techniques applied in Supply Chain—Traceability and Transparency studies. Both regression and NN approaches are represented equally, with one study each, suggesting that researchers are exploring a mix of traditional predictive models and more flexible DL approaches.

As illustrated in Figure 9B, supervised learning accounts for 100% of the reviewed publications in this category. The absence of unsupervised or hybrid methods indicates a strong reliance on labeled datasets, likely driven by the structured nature of supply chain and traceability applications.

In terms of task type, Figure 9C highlights that all studies formulate their problems as classification tasks. This reflects the emphasis on categorical decision-making in supply chain contexts, such as determining product authenticity, verifying traceability, or classifying transparency levels, rather than continuous regression-based predictions.

Overall, these findings reveal a methodological focus on supervised classification approaches, with both regression models and NNs contributing equally to supply chain and traceability applications.

### 3.8. Food Industry Efficiency and Industry 4.0 Models

Finally, the review synthesizes applications of ML within the broader context of Industry 4.0—including autonomous QC monitoring, hybrid mechanistic-ML modeling, and the convergence with big data analytics, robotics, and digital twins. Methodological trends, task typologies, and sensor/ML integration frameworks are mapped across figures and comparative tables.

Rakholia et al. [36] developed an ML–based system to predict drying times for meat-based food products in a smart manufacturing environment, with the goal of improving resource allocation, production planning, and sustainability. The study integrated Enterprise Resource Planning (ERP) data (product composition, mass, and process details) with Supervisory Control and Data Acquisition (SCADA) sensor data like temperature, humidity, fan speed, and heating/cooling status collected at 10 s intervals via an IoT network. After preprocessing steps such as outlier removal, missing value imputation, and aggregation, the authors implemented an XGBoost ensemble regressor due to its robustness with limited datasets and ability to model nonlinear relationships. The model was trained on data from four high-frequency product IDs (≥300 records each) and evaluated via a sliding window approach (60-day window, 20-day shift), with hyperparameter tuning by random search. Model performance was evaluated using RMSE, MAE, and MAPE. Additionally, SHAP-based explainable AI was employed to identify the key drivers influencing model predictions, revealing that environmental parameters had a greater impact than product composition. The model demonstrated high predictive accuracy and strong generalizability, achieving an RMSE of 47.14 min, an MAE of 36.27 min, and a MAPE of 0.56%. In a related study on meat quality assessment, Rakholia et al. [36] applied a similar AI-driven drying-time prediction framework, integrating process-sensor data with regression models, and achieved an 18% reduction in prediction error compared to baseline methods—further underscoring the potential of such systems for resource optimization and production planning in smart manufacturing.

Konur et al. [46] presented a case study on transforming a traditional small–medium-sized enterprise (SME) food manufacturer, operating century-old equipment, into an Industry 4.0-enabled smart factory without replacing existing machinery. The study developed and deployed a smart production control system integrating big data analytics, IoT sensors, ML, cyber-physical systems, and cloud computing to improve product consistency, production efficiency, and operational decision-making. Data were collected from oven temperature profiles, environmental sensors, and operational parameters like cutter and conveyor speeds, generating over 250,000 data instances in six months. The system applied supervised ML models. In particular, a KNN classifier developed to predict optimal baking conditions, achieved the highest training accuracy (98.8%) and prediction accuracy (94.7%) compared to Logistic regression, Naïve Bayes, MLP, and SVM variants. The solution reduced production variability, lowered energy costs, and increased capacity, while providing real-time monitoring, decision support dashboards, and virtualized factory control. The approach serves as a reference architecture for Industry 4.0 adoption in SMEs with legacy infrastructure.

Redchuk et al. [50] presented an Industry 5.0 case study in a North American food ingredient company, demonstrating how a Low-Code Platform (LCP) integrated with an Industrial Internet of Things (IIoT) infrastructure and ML could optimize boiler thermal efficiency to reduce fuel consumption and carbon emissions. Using 18 months of historical process and ambient data like boiler pressure, air flow, fuel input, inlet water temperature, ambient temperature, humidity, and wind speed/direction, the team configured three ML models via the LCP’s pre-built templates: (1) boiler simulator model to predict fuel usage, (2) optimal control parameters model to minimize total fuel use while meeting steam demand, and (3) fuel consumption model to identify top drivers. The methodology followed Lean Startup’s Build–Measure–Learn cycle, involving operators and engineers in model co-creation to ensure domain relevance. Models were deployed to Microsoft Azure Cloud for real-time integration with the IoT platform via Application Programming Interface (API). Testing showed a 2.5% improvement in boiler thermal performance, a 4% reduction in fuel costs, and an annual reduction of over 10 million pounds of CO_2_ emissions. The approach reduced implementation time, improved operator engagement, and delivered actionable AI-driven recommendations without high coding requirements.

Vargas et al. [51] implemented a hybrid Lean Six Sigma (LSS) following the define, measure, analyze, improve, control (DMAIC) methodology integrated with a Surface Tension Neural Network (STNN) to optimize garlic salt production in a condiment SME. The STNN classified mill temperature and product humidity in real-time, enabling precise process control and waste reduction. IoT-based sensors with a Wi-Fi module fed temperature and humidity data to the STNN, which was deployed on Google Cloud IoT. Compared to a Naive Bayes (NB) classifier, the STNN achieved higher accuracy for both temperature (97.10%) and humidity (97.31%) classification. Post-implementation, the process yield increased from 94.22% to 97.36% (+3.14%), waste was reduced by 39.7 kg per batch, sigma level improved by +2.13 points, and defects per million opportunities (DPMO) decreased by 551.722. Economic savings amounted to USD 158.5 per batch. This study demonstrated that integrating AI-driven real-time classification with LSS can significantly enhance operational efficiency, quality consistency, and sustainability in SME food manufacturing.

Liu et al. [52] empirically examined the impact of AI adoption on firm productivity and performance in China’s food processing manufacturing industry, focusing on the mediating roles of labor skill structure and total factor productivity (TFP). Using a panel dataset of 194 listed food processing companies (1702 firm-year observations) from 2010 to 2021, the authors constructed enterprise-level AI adoption indicators via text mining of annual reports, identifying the first year of AI implementation through keywords such as “AI” and “intelligent production line.” A multi-way fixed effects regression model was applied to assess AI’s effect on return on assets (ROA), alongside robustness checks, heterogeneity analysis, and channel mechanism testing. Results indicated that AI adoption significantly improved firm performance, primarily through increasing the proportion of high-skilled labor and enhancing TFP, with stronger effects observed in state-owned enterprises, capital-intensive firms, and companies located in China’s western region. Interaction effects showed that AI combined with higher-skilled labor or higher TFP further amplified performance gains. The study concludes that targeted investment in skilled human capital and AI-enabled productivity improvements can substantially enhance competitiveness in the food processing sector.

A detailed summary of the reviewed works, including data size, modeling methods, and evaluation metrics, is presented in Table 7.

#### Overview of Food Industry Efficiency and Industry 4.0 Models

**Figure 10:**
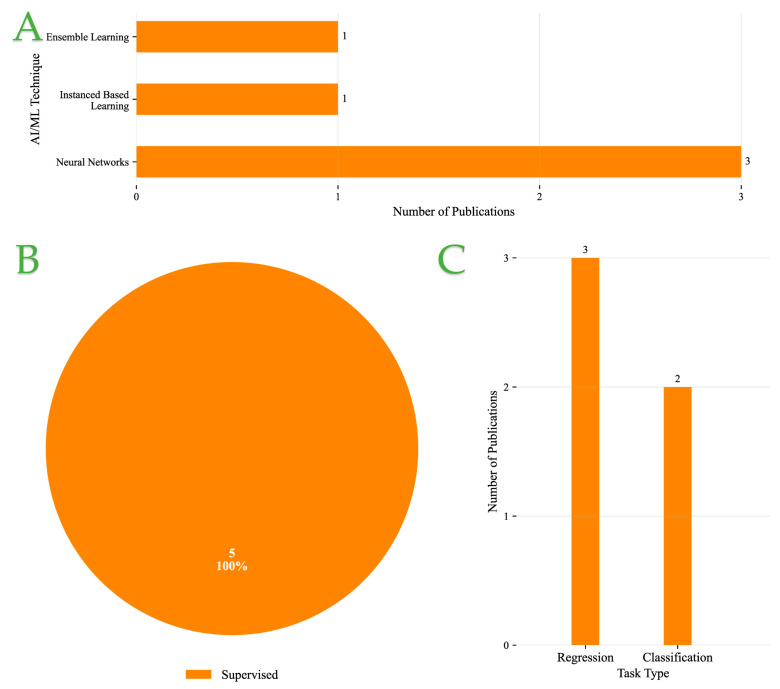
(**A**) Distribution of AI/ML techniques in Food Industry Efficiency and Industry 4.0 Models category. (**B**) Types of learning approaches used in Food Industry Efficiency and Industry 4.0 Models category. (**C**) Prediction task in Food Industry Efficiency and Industry 4.0 Models category.

Figure 10A illustrates the distribution of AI/ML techniques applied in the context of Food Industry Efficiency and Industry 4.0 Models. NNs are the most prominent, appearing in three studies, showcasing their strong applicability for modeling complex industrial processes. Ensemble learning and instance-based learning methods are each used in one study, reflecting a more limited but targeted adoption in specialized scenarios.

As shown in Figure 10B, all reviewed publications in this category rely on supervised learning (100%). The absence of unsupervised or hybrid methods highlights the dominant reliance on labeled datasets in industrial efficiency studies, where well-defined input–output relationships are crucial for performance optimization.

Regarding task formulation, Figure 10C indicates a balance between regression and classification, with three and two studies, respectively. Regression tasks emphasize the prediction of continuous efficiency metrics (e.g., energy use, throughput, cost), while classification tasks address categorical decision-making such as fault detection or process state identification.

Overall, these findings reveal a methodological preference for supervised NN-based approaches, with both regression and classification tasks playing complementary roles in optimizing Food Industry Efficiency and advancing Industry 4.0 applications.

## 4. Conclusions and Future Outlook

**Figure 11:**
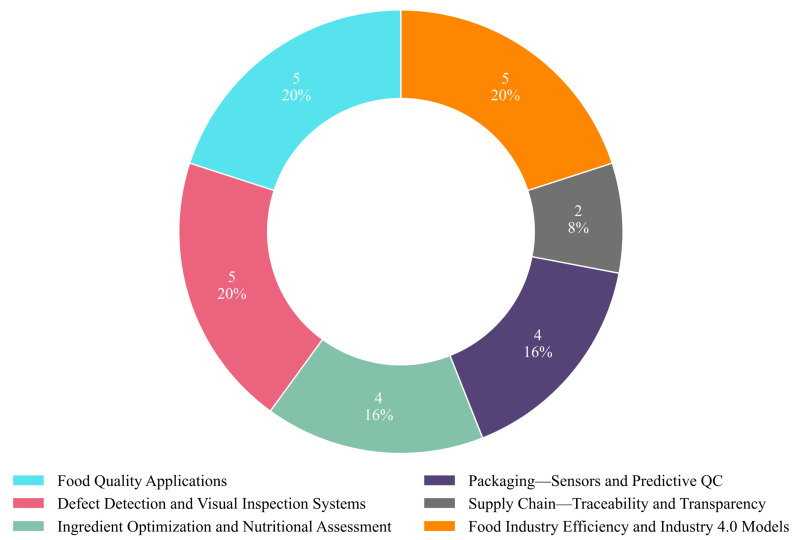
Pie chart presenting the approaches according to each category.

In this review, we systematically analyzed the role of ML and AI-driven QC systems in enhancing QC processes across the food industry. Thee selected publications were categorized based on application domain, AI/ML technique, learning paradigm, and task objective. As shown in Figure 11, 6 major application areas were identified across the 25 reviewed studies. The most represented categories were Food Quality Applications, Defect Detection and Visual Inspection Systems, and Food Industry Efficiency and Industry 4.0 Models, each comprising 20% of the total (five publications each). These were followed by Ingredient Optimization and Nutritional Assessment and Packaging—Sensors and Predictive QC, both accounting for 16% (four publications each). The least represented category was Supply Chain—Traceability and Transparency, with 8% (two publications). This distribution reflects a strong focus on product-centric AI applications, while supply chain-related innovations remain relatively underexplored.

**Figure 12:**
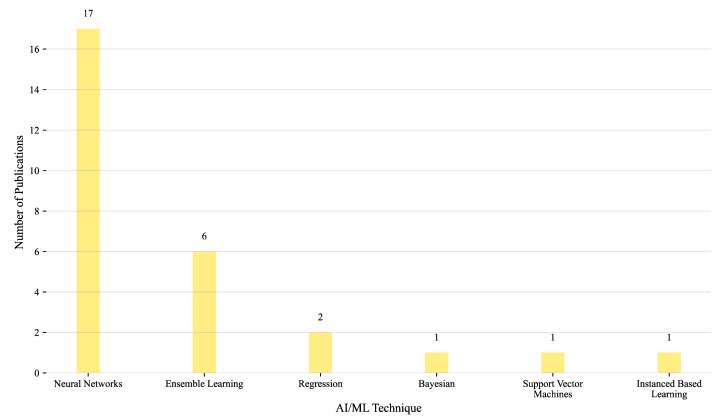
Distribution of AI/ML techniques.

A clear methodological trend emerges with NNs dominating the landscape, appearing in 17 of the reviewed studies. Their widespread use underscores their strong suitability for modeling complex nonlinear relationships and high-dimensional data patterns across food-related applications. Ensemble learning methods are the second most common approach, applied in six studies, suggesting moderate interest in leveraging combined model strategies for improved robustness. Regression-based techniques are used in two studies, while Bayesian methods, SVMs, and Instance-Based Learning each appear in only one study. This distribution, illustrated in Figure 12, highlights the dominant reliance on neural architectures for AI/ML tasks in the food sector, reflecting their predictive power and flexibility. However, the underrepresentation of alternative methods also suggests a lack of comprehensive benchmarking and limited exploration of techniques that may offer advantages in terms of interpretability, computational efficiency, or uncertainty modeling.

**Figure 13:**
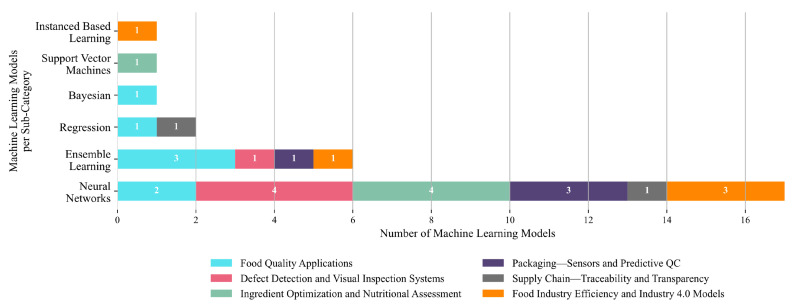
Distribution of ML techniques across methodological categories.

According to the application domain, NNs are the most dominant technique, accounting for 16 out of 27 model applications (59.3%) (Figure 13). They are applied across all six sub-domains, with the highest concentration in Defect Detection and Visual Inspection Systems and Ingredient Optimization and Nutritional Assessment (four studies each, 14.8%), followed by Packaging—Sensors and Predictive QC and Industry 4.0 Models (three studies each, 11.1%) and Food Quality Applications (two studies, 7.4%). Ensemble learning represents the second most frequently used approach, appearing in six studies (22.2%). These are distributed across Food Quality Applications (three studies, 11.1%) and one study each (3.7%) in Defect Detection and Visual Inspection Systems, Packaging and Predictive QC, and Industry 4.0 Models. Other techniques are used sparingly. Regression models account for two studies (7.4%), appearing in Ingredient Optimization and Supply Chain categories. Bayesian methods, SVMs, and Instance-Based Learning are each represented in only one study (3.7%). These are applied in Packaging and Predictive QC, Ingredient Optimization, and Industry 4.0 Models, respectively. Overall, the data reflect a clear methodological preference for neural networks, with ensemble models forming a secondary but broadly distributed approach. The limited presence of alternative algorithms suggests a gap in comparative analysis and highlights the need for broader exploration of models that may offer interpretability, efficiency, or probabilistic insights in food industry applications.

**Figure 14:**
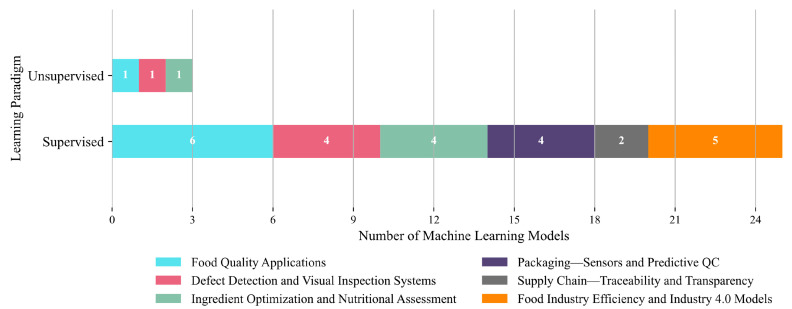
Distribution of learning types across methodological categories.

In terms of learning paradigm, supervised learning overwhelmingly dominates, with 23 out of 26 model applications (88.5%) employing it across all sub-domains. It is used exclusively in Food Industry Efficiency and Industry 4.0 Models (five studies, 19.2%), Packaging—Sensors and Predictive QC (four studies, 15.4%), and Supply Chain—Traceability and Transparency (two studies, 7.7%). It also represents the majority in Food Quality Applications (six studies, 23.1%), Defect Detection and Visual Inspection Systems (four studies, 15.4%), and Ingredient Optimization and Nutritional Assessment (four studies, 15.4%). In contrast, unsupervised learning appears only in three studies (11.5%), with one instance each in Food Quality Applications, Defect Detection and Visual Inspection Systems, and Nutritional Assessment. Its marginal presence suggests that although there is growing awareness of its utility for unlabeled or exploratory analysis, the field continues to rely heavily on labeled datasets and deterministic supervised workflows. This imbalance reflects both the maturity and perceived reliability of supervised learning pipelines, as well as a lack of experimentation with alternative paradigms. Figure 14 thus highlights a clear methodological leaning, while also pointing to untapped potential in unsupervised and hybrid approaches within food systems research.

**Figure 15:**
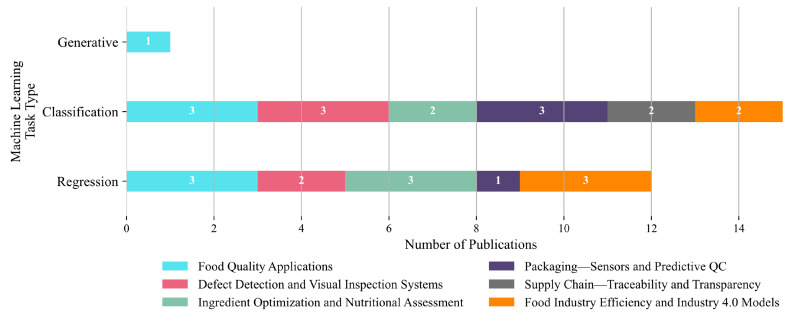
Contribution of methodological categories per ML task type.

Classification emerges as the most common task type, appearing in 13 out of 26 studies (50%). It spans all application domains, including Food Quality Applications (three studies), Defect Detection and Visual Inspection Systems (three), Ingredient Optimization and Nutritional Assessment (three), Packaging—Sensors and Predictive QC (three), Supply Chain—Traceability and Transparency (two), and Food Industry Efficiency and Industry 4.0 Models (two). Its widespread use reflects its essential role in categorical decision-making processes, such as defect detection, sorting, and system status identification. Regression follows closely, with 12 studies (46.2%), primarily concentrated in Food Quality Applications (3 studies), Ingredient Optimization and Nutritional Assessment (3), Defect Detection and Visual Inspection Systems (2), Packaging and Predictive QC (1), and Food Industry Efficiency and Industry 4.0 Models (3). These use cases highlight regression’s importance in estimating continuous variables such as nutritional values, process parameters, or efficiency metrics. Generative modeling appears only once (3.8%), exclusively within Food Quality Applications, indicating its nascent stage in this field. Overall, Figure 15 illustrates a balanced methodological reliance on both regression and classification tasks to address predictive and categorical needs across food industry domains. The near absence of generative approaches suggests a promising area for future exploration, particularly in data augmentation, simulation, and design automation contexts.

Several critical insights and future directions emerge from this analysis. Firstly, explainability and transparency remain under-addressed, particularly in DL-based systems. Integrating explainable AI (XAI) techniques such as SHAP and LIME could significantly enhance interpretability, particularly in regulatory-sensitive domains like food safety, enabling human–machine trust and diagnostic traceability. Secondly, data scarcity continues to hinder model generalizability, especially in cases involving 3D packaging, spoilage detection, and emerging product formulations. Investments in shared, open datasets, synthetic data generation, and semi-supervised approaches will be pivotal in overcoming this challenge.

Thirdly, cross-domain integration between smart packaging, supply chain analytics, and sensor-based inspection remains limited. End-to-end ML pipelines—incorporating traceability, predictive maintenance, and real-time quality monitoring—could enable closed-loop automation and transparency in food manufacturing. Lastly, evaluation rigor varies considerably across studies, with inconsistent reporting of metrics, validation protocols, and deployment feasibility. The adoption of standardized benchmarking protocols, lifecycle-oriented evaluation, and sustainability-driven KPIs will be essential for industrial readiness.

Looking ahead, several promising research and development directions emerge:

- Multi-modal integration for richer, more resilient QC systems that combine visual, sensor, and contextual data;
- On-device/edge deployment for real-time, low-latency decision-making, reducing dependence on high-bandwidth connections;
- Scalable, open datasets and benchmarking platforms to accelerate reproducibility and cross-domain comparisons;
- Reinforcement and adaptive learning for dynamic process control in rapidly changing manufacturing conditions;
- Sustainability-oriented design, optimizing QC not only for defect minimization but also for waste reduction, energy efficiency, and circular economy goals.

In conclusion, the application of ML in food QC is a rapidly maturing field marked by strong advances in visual inspection, quality prediction, and ingredient optimization. However, the methodological base remains narrow, with an over-reliance on supervised paradigms and NNs. Advancing the field will require methodological diversification, transparent and interpretable systems, improved data infrastructure, and system-level optimization frameworks—enabling not just operational excellence but measurable contributions to industrial sustainability and democratized access to advanced QC technologies.

This review does not include the field of predictive food microbiology, which is recognized as an important and rapidly developing area for food safety and quality control. However, it was not part of the original scope and objectives of the study and therefore was not incorporated into the main analysis. Additionally, the review is limited to studies indexed in Scopus and published in English, which may have excluded relevant work in other databases or languages. The predominance of imaging-focused studies in the included literature also reflects a current bias in the field, and some application domains remain underrepresented in large-scale industrial validation.

While this review covers multiple product domains—including produce, meat, dairy, beverages, packaging, and supply chain—the depth of discussion within each category is necessarily limited by the broad scope of the work. The primary aim was to identify cross-cutting trends, methodological patterns, and research gaps rather than to provide exhaustive coverage of any single domain. In real-world deployment, ML-based QC systems face several challenges: (i) data scarcity and variability across production environments, (ii) limited model generalizability when moving from lab to factory settings, (iii) integration with legacy equipment and workflows, (iv) compliance with regulatory and food safety standards, and (v) cost–benefit trade-offs in scaling advanced sensing and computing infrastructure. Addressing these issues will be critical for translating research prototypes into robust, industry-ready solutions.

The novelty of this review lies in its combination of a transparent PRISMA-based methodology, a six-domain thematic framework enabling cross-domain insights, and the integration of Industry 4.0/5.0 innovations into the food QC context. By aligning metrics and identifying methodological and domain gaps, it provides greater systematic depth and actionable guidance than prior reviews.

## Figures and Tables

**Table 2:** Summary of studies in Food Quality Applications category.

Cite	Data Size	Observed Features	AI Method	Evaluation	Results
[19]	141 samples from 3 sweet potato varieties	VNIR-HSI spectra 400–1000 nm, DMC, SSC and firmness	Supervised PLSR regressor	R^2^, RMSEP, RPD	DMC: R^2^ = 0.92 RMSEP = 1.50 RPD = 3.58 SSC: R^2^ = 0.66 RMSEP = 0.85 RPD = 1.72 Firmness: R^2^ = 0.85, RMSEP = 1.66 RPD = 2.63
[32]	24 batches of Eucommiae cortex samples	Metabolite profiles from Liquid Chromatography–Mass Spectrometry (LC–MS); color features from digital images, red, green, blue, L*, a*, b*, L, ΔΕ	Supervised XGBoost regressor	R^2^	R^2^ = 0.83
[37]	1800 grayscale images 512 × 512 px	Rawhide stick defect images, surface stains, irregular shapes	Unsupervised DCGAN generator and Supervised CNN classifier	IS, FID, SSIM	IS = 6.12, FID = 105.61 SSIM = 0.72
[41]	Not specified (IoT sensor data from milk spoilage monitoring)	Temperature, pH, gas concentration readings	Supervised RF classifier	Accuracy	Accuracy = 0.98
[42]	Wheat: 63 samples, Dairy: 1059 samples, Wine: 6497 samples—1599 red and 4898 white	Wheat: physicochemical and biochemical properties Dairy: pH, temperature, taste, odor, fat, turbidity, color Wine: physicochemical composition	Supervised RF regressor and classifier and Supervised BN–SCM regressor	R^2^, MSE, PE ACE	Wheat: R^2^ = 0.75 Protein ACE = 0.65 Total high molecular-mass (THMM) ACE = 0.42 Dairy: MSE < 1.00% Temperature ACE = −0.04 Fat ACE = 0.40 Wine: PE = 5.13% (red) PE = 4.14% (white) Alcohol ACE = 0.35

RMSEP is in % for DMC of the sample’s mass, RMSEP for SSC is in degrees °Bx, which is the sugar content in an aqueous solution, RMSEP for firmness is in Newtons (N), representing the force required to deform the sample. L*, a*, b*: CIELAB color coordinates (L* = lightness, a* = green–red axis, b* = blue–yellow axis). Note: The reported metrics are task-specific and should not be interpreted as directly comparable across studies.

**Table 3:** Summary of studies in Defect Detection and Visual Inspection Systems category.

Cite	Data Size	Observed Features	AI Method	Evaluation	Results
[38]	Number of chocolate samples not specified, multiple shapes (e.g., circular, square, triangular) and oleogel formulations (e.g., monoglycerides, sucrose fatty acid ester, HPMC at 1–3%)	Shape geometry, contour features, extrusion completeness, height uniformity	Supervised ANN classifier	Accuracy	Shape recognition: Circular shapes ≈ 90%, Square/triangular shapes = ~70–85% Defect detection: Major defects > 90%, Subtle defects < 50%
[14]	High-resolution RGB images from four reusable cup categories: 245 samples	Color, texture, and shape patterns	Unsupervised ANN autoencoder classifier	ROC–AUC, F1-score	Image-level: ROC–AUC = 0.96, F1-score = 0.97 Pixel-level: ROC–AUC = 0.92, F1-score = 0.51
[43]	Wine quality: 6800 Portuguese wine samples—1900 red and 4900 white, Wine origin: 178 Italian wine samples, Olive oil origin: 572 samples	11 physicochemical variables with quality score, 13 chemical characteristics, 7 fatty acid variables	Supervised RF regressor	Accuracy, ROC–AUC	Wine quality: Accuracy = 0.63 for white, Accuracy = 0.65 for red, ROC–AUC = 0.86 for white, ROC–AUC = 0.77 for red Wine origin: Accuracy = 1.00, ROC–AUC = 1.00 Olive oil origin: Accuracy = 0.96, ROC–AUC = 1.00
[47]	Egg images: 794, 632 damaged and 162 intact	Egg surface color and texture patterns from RGB images under varied backgrounds, 960 × 1280 px	Supervised CNN classifier	Accuracy, Precision, Recall, F1-score, ROC–AUC	Accuracy = 0.98, Precision = 0.98, Recall = 1.00, F1-score = 0.99, ROC–AUC = 0.94
[48]	Gummy candy images: 20,000 augmented from 5000 originals	Color, shape, and texture features from RGB images	Supervised CNN classifier	Precision, Recall, F1-score	Precision = 0.93, Recall = 0.87, F1-score = 0.90

Note: The reported metrics are task-specific and should not be interpreted as directly comparable across studies.

**Table 4:** Summary of studies in Ingredient Optimization and Nutritional Assessment category.

Cite	Data Size	Observed Features	AI Method	Evaluation	Results
[34]	84 experimental runs were conducted, corresponding to 28 frying-condition combinations performed in triplicate	Temperature, frying time, oil amount	Supervised ANN regressor	R^2^, RMSE	PUFA/SFA: R^2^ = 0.99, RMSE = 0.038 IA R^2^ = 0.98, RMSE = 0.046
[35]	Formulations × triplicate: 93 samples	Ingredient concentrations, GWP, cost, growth rate	Supervised ANN regressor	RMSE, MAPE	RMSE = 0.01, MAPE = 0.90
[39]	30 experimental runs	Concentrations of salad peel waste hydrolysate, malt extract, CaCl_2_, pH	Supervised SVM regressor	R^2^	R^2^ = 0.9772
[49]	FoodSeg103: 7118 images, UECFoodPix Complete: 10,000 images	Ingredient-level color, texture, and shape features from food images	Unsupervised DNN classifier and Supervised ANN classifier	mIoU	FoodSeg103: Bread = 0.69, Carrot = 0.67, Chicken = 0.56, Sauce = 0.54, Tomato = 0.58 UECFoodPix Complete: Salad = 0.73, Beverage = 0.75, Soup = 0.72, Noodle = 0.72, Rice = 0.76

Note: The reported metrics are task-specific and should not be interpreted as directly comparable across studies.

**Table 5:** Summary of studies in Packaging—Sensors and Predictive QC category.

Cite	Data Size	Observed Features	AI Method	Evaluation	Results
[33]	3 treatment groups × 3 DBD times × 5 replicates × 12 days	Mass loss, pH, SSC, TA, hue angle, chroma, bioyield stress	Supervised ANN regressor	R^2^, RMSE	CO_2_: R^2^ = 0.98, RMSE = 0.47 Ethylene: R^2^ = 0.93, RMSE = 5.37
[40]	Not specified; simulated and comparative data	Packaging material properties, structural design variables, energy usage metrics	Supervised ANN classifier	Accuracy	Accuracy = 0.97
[24]	80,000 images of 100 packaged food types	Packaging design features under varied lighting, angles, and framing	Supervised CNN classifier	Precision, Recall, F1-score, Accuracy	Precision = 0.99, Recall = 1.00, F1-score = 0.99, Accuracy = 0.98
[22]	497 vibration segments from 13 experiments	Raw, specialized kurtosis, generic features	Supervised RF classifier	Accuracy	Generic features: Accuracy = 1.00 Specialized kurtosis: Accuracy = 0.88 Raw: Accuracy = 0.83

Note: The reported metrics are task-specific and should not be interpreted as directly comparable across studies.

**Table 6:** Summary of studies in Supply Chain—Traceability and Transparency category.

Cite	Data Size	Observed Features	AI Method	Evaluation	Results
[44]	Not explicitly mentioned	Temperature, humidity, CO_2_, chemical profiles, isotopic ratios	Supervised PLS-DA classifier	Accuracy	0.98
[45]	2469 food waste	Not explicitly mentioned	Supervised CNN classifier	F1-score	0.96

Note: The reported metrics are task-specific and should not be interpreted as directly comparable across studies.

**Table 7:** Summary of studies in Food Industry Efficiency and Industry 4.0 Models category.

Cite	Data Size	Observed Features	AI Method	Evaluation	Results
[36]	ERP, SCADA, IoT sensor data over 15 months and 4 high-frequency product identification	Product composition, product mass, temperature, humidity, fan speed, heating/cooling status, drying room characteristics	Supervised XGBoost regressor	R^2^, RMSE, MAE, MAPE	R^2^ = 0.96, RMSE = 47.14 min, MAE = 36.27 min, MAPE = 0.0056
[46]	>250,000 temperature and operational data points over 6 months	Oven zone temperatures, environmental variables, cutter and conveyor speeds, baking quality scores	Supervised KNN classifier	Accuracy	Accuracy = 0.98
[50]	18 months of IIoT process and ambient data from multiple boilers and turbines	Boiler pressure, boiler air flow, boiler fuel input, boiler air input temperature, inlet water supply temperature, ambient temperature, humidity, wind speed, wind direction	Supervised ANN regressor	ML evaluation metric not reported	Achieved 2.5% improvement in thermal performance, 4% reduction in fuel costs, >10 million pounds/year CO_2_ reduction
[51]	IoT sensor data from garlic salt production batches in a condiment SME	Mill process temperature, garlic salt product relative humidity	Supervised ANN classifier	Accuracy	Humidity: Accuracy = 0.97 Temperature: Accuracy = 0.97
[52]	1702 firm-year observations	ROA, TFP, OP, labor skill structure, ownership type, leverage, growth, cash flow, firm age, region, factor intensity	Supervised ANN regressor	R^2^	ROA: R^2^ = 0.008 (*p* < 0.1) TFP: R^2^ = 0.042 (*p* < 0.01) Labor: R^2^ = 0.042 (*p* < 0.01) Region: R^2^ = 0.024 (*p* < 0.05)

Note: The reported metrics are task-specific and should not be interpreted as directly comparable across studies.

## Data Availability

No new data were created or analyzed in this study.

## Abbreviations

The following abbreviations are used in this manuscript:

ACE	Average Causal Effect
AI	Artificial Intelligence
AIoT	Artificial Intelligence of Things
ANN	Artificial Neural Network
BN	Bayesian Network
BN–SCM	Bayesian Network—Structural Causal Model
CCD	Central Composite Design
CNN	Convolutional Neural Network
DMC	Dry Matter Content
DNN	Deep Neural Network
GAN	Generative Adversarial Network
GBM	Gradient Boosting Machine
GMP	Good Manufacturing Practices
GWP	Global Warming Potential
HACCP	Hazard Analysis and Critical Control Points
HAM	Hybrid Attention Mechanism
HPMC	Hydroxypropyl Methylcellulose
HSI	Hyperspectral Imaging
IA	Index of Atherogenicity
IIoT	Industrial Internet of Things
IoT	Internet of Things
ISO	International Organization for Standardization
KNN	k-Nearest Neighbors
LSS	Lean Six Sigma
MAP	Modified Atmosphere Packaging
MAPE	Mean Absolute Percentage Error
mIoU	Mean Intersection over Union
ML	Machine Learning
MLP	Multi-Layer Perceptron
NSGA-II	Non-dominated Sorting Genetic Algorithm II
OP	Operating Profit
PLSR	Partial Least Squares Regression
QC	Quality Control
RF	Random Forest
RMSE	Root Mean Square Error
RMSEP	Root Mean Square Error of Prediction
RPD	Ratio of Prediction to Deviation
RSM	Response Surface Methodology
SCM	Structural Causal Model
SHAP	SHapley Additive exPlanations
SME	Small and Medium-sized Enterprise
SVM	Support Vector Machine
SVR	Support Vector Regression
SSC	Soluble Solid Content
STNN	Surface Tension Neural Network
TFP	Total Factor Productivity
VNIR-HSI	Visible and Near-Infrared Hyperspectral Imaging
XAI	Explainable Artificial Intelligence
XGBoost	Extreme Gradient Boosting
YOLO	You Only Look Once
